# Sustained relief of chronic headache using percutaneous 60-day peripheral nerve stimulation treatment: 12-month results from a multicenter, prospective clinical study

**DOI:** 10.1016/j.inpm.2026.100806

**Published:** 2026-07-30

**Authors:** Zachary L. McCormick, Christopher A. Gilmore, Genaro G. Gutierrez, Mitchell P. Engle, Matthew J. Pingree, Jason E. Pope, David J. DiBenedetto, Narayan R. Kissoon, William J. Huffman, Nathan D. Crosby, Joseph W. Boggs

**Affiliations:** a Department of Physical Medicine and Rehabilitation, University of Utah School of Medicine, Salt Lake City, UT, USA; b Center for Clinical Research, Winston-Salem, NC, USA; c Pain Specialists of America, Austin, TX, USA; d Institute of Precision Pain Medicine, Corpus Christi, TX, USA; e Anesthesiology and Perioperative Medicine, Mayo Clinic, Rochester, MN, USA; f Evolve Restorative Center, Santa Rosa, CA, USA; g MedVadis Research, Waltham, MA, USA; h SPR, Cleveland, OH, USA

**Keywords:** 60-day PNS, Cervicogenic headache, Headache, Occipital nerves, Occipital neuralgia, Peripheral nerve stimulation

## Abstract

**Objective:**

The study aimed to assess the long-term durability of pain relief among patients with cervicogenic headache (CGH) or occipital neuralgia (ON) following percutaneous 60-day peripheral nerve stimulation (PNS) treatment.

**Background:**

Cervicogenic headache (CGH) and occipital neuralgia (ON) are challenging headache conditions with significant impact on function and quality of life. The present feasibility study initially reported that percutaneous 60-day PNS can reduce headache, facilitating functional improvements such as improved quality of life and reduced neck disability. This work evaluates the durability of clinical outcomes through 12 months within the study cohort.

**Methods:**

Twenty participants with CGH or ON (and without migraine) received bilateral, percutaneous leads stimulating the occipital nerves. Leads were left indwelling for up to 60 days before being withdrawn. During prospectively defined follow-up through 12 months, participants reported on their occipital pain intensity and pain interference, as well as secondary outcomes.

**Results:**

Through 12 months, 82% of participants reported sustained, substantial reductions (≥50%) in pain and/or pain interference. Clinically meaningful changes in secondary outcomes, including pain-related neck disability, impact of headache on daily life, and quality of life, were also observed. Results through 3 months on device effectiveness and safety have been previously published. No new device-related adverse events were reported in the follow-up phase of the study among these participants.

**Conclusions:**

A majority of participants with CGH or ON had sustained relief from pain and pain interference, resulting improvements in function, and reductions in disability through 12 months following a percutaneous 60-day PNS treatment without any new device-related adverse events reported after the 3-month follow-up. These findings support the feasibility for 60-day PNS in the treatment of refractory pain due to CGH or ON.

## 1 Introduction

Cervicogenic headache (CGH) and occipital neuralgia (ON) are headache conditions that are challenging to treat, diminish quality of life, and are associated with significant healthcare utilization [1,2]. Both conditions are characterized, in part, by severe pain in the distribution of the occipital nerves, making interventions targeting the occipital nerves (e.g., occipital nerve block, ONB) commonly considered treatment options if patients do not respond to other conservative measures such as pain medications [1,3]. While interventions like ONBs may provide meaningful relief, the effects are typically short-lived [4], leading to persistent pain, negative impact on function and quality of life, as well as additional healthcare utilization and associated costs.

Peripheral nerve stimulation (PNS) targeting the occipital nerves has shown promise as an effective treatment for certain headache disorders, with long-term benefits reported in studies using conventional permanently implanted systems [5,6]. However, historic use of PNS for treatment of headache conditions utilized systems not intended to be implanted in the posterior head and neck region, resulting in device-related complications, high rates of surgical revision during follow-up, and diminished therapeutic benefit [[7], [8], [9], [10]]. For example, conventional epidural spinal cord stimulation leads demonstrated susceptibility to lead fracture, erosion, and migration when implanted in the highly mobile head and neck region [8,11].

One novel, FDA-cleared PNS device may address these challenges through a design intended to resist lead migration and fracture even in highly mobile regions of the body including the head and neck. This percutaneous 60-day PNS system has demonstrated efficacy in multiple prospective studies for various chronic pain conditions in similarly demanding body regions including highly mobile joints such as the shoulder and knee [12,13]. Follow-up through up to 12 months in previous studies with other chronic pain conditions also consistently demonstrates the potential for patients to experience durable pain relief extending beyond the 60-day treatment period [[14], [15], [16]]. Additionally, successful utilization of the percutaneous 60-day PNS system for treatment of headache has been reported in retrospective case reports and case series [[17], [18], [19]].

The present prospective, multicenter, single-arm feasibility study was conducted to investigate the effectiveness of this treatment among participants with occipital headache due to CGH and ON. As reported previously, participants had substantial reductions in pain and pain interference through 3 months, facilitating improvements in other domains including physical function and quality of life [20]. This report evaluates follow-up clinical outcomes in the same study cohort through 12 months to assess the durability of pain relief and improvement in other domains of health.

## 2 Methods

This multicenter, prospective, single-arm feasibility study was approved on ClinicalTrials.gov (NCT05491915) and by the WCG Internal Review Board (WIRB) and local IRBs (as applicable) for participating sites. The goal of this study was to evaluate the feasibility of lead implantation targeting the occipital nerves with 60-day PNS used for the treatment of CGH or ON with regard to both effectiveness and safety. An earlier publication described the results of this study through the 3-month follow-up [20]. Here, data on the effectiveness and safety of percutaneous 60-day PNS are presented through the 12-month follow-up.

### 2.1 Eligibility criteria

Participants were required to have a prior diagnosis of CGH or ON (e.g., in accordance with the third edition of the International Classification of Headache Disorders [ICHD-3] criteria) present in their health records with an average pain intensity score ≥4/10 (Brief Pain Inventory Short Form [BPISF] Question #5, assessed using a week-long pain diary) and average pain interference score ≥4/10 (BPISF Question #9) that cannot be attributed to cancerous lesion or a headache condition other than CGH or ON, including tension-type headache, analgesic medication over-use or withdrawal headache, cluster headache, or vascular and non-vascular intracranial disorders. Additional key eligibility criteria included age ≥18 years, a lack of migraine diagnosis and migraine-like features (assessed using the ID Migraine Screener, score ≤1), Beck Depression Inventory II (BDI-II) score of ≤20, no prior surgery in the cranial or upper cervical area, stable pain medications for ≥4 weeks prior to PNS treatment onset, and sufficient wash-out periods for other interventions targeting cranial or upper cervical structures: no corticosteroid injection in the last 6 weeks, no botulinum toxin injection in the last 3 months, and no radiofrequency ablation in the last 6 months. Participants provided written informed consent before completing baseline assessment surveys and a baseline 7-day diary to measure average occipital pain intensity, headache events, and pain medication use.

### 2.2 Occipital nerve block

To assess the potential relationship between occipital nerve block (ONB) response and percutaneous 60-day PNS treatment response, all participants without records of qualifying ONB from the prior 3 months underwent a diagnostic ONB (using a local anesthetic without corticosteroids; lidocaine, bupivacaine, or a combination of the two) targeting the greater and third occipital nerves at least 7 days before PNS lead implantation and reported pain reduction using a pain diary. The presence or lack of response to ONB did not affect a participant's eligibility, and their diagnosis of CGH or ON was not reevaluated based on ONB response. The results of the ONB were highlighted in the initial study publication [20]. All participants received at least partial relief from pain following ONB, and mean weekly pain scores obtained before the ONB and at the time of lead implantation were comparable (5.6 and 5.5, respectively), which could indicate that pain levels returned approximately to baseline levels prior to the start of PNS treatment.

### 2.3 Percutaneous 60-day peripheral nerve stimulation

Participants underwent bilateral implantation of leads targeting the greater and third occipital nerves using a commercially available, percutaneous 60-day PNS system (SPRINT ® PNS System, SPR, Cleveland, OH, USA). Participants received bilateral leads to standardize the PNS treatment and account for potential unmasking of contralateral pain that could occur during unilateral treatment. All PNS leads were implanted under fluoroscopic and/or ultrasound guidance. Leads were percutaneously implanted typically using an insertion site at the C4 or C5 level with the active electrode tip positioned at the posterior aspect of the C2 lamina cephalad to the C2/3 joint. The implanting physician optimized lead position using stimulation-evoked sensations as reported by the participant, with the goal of producing comfortable sensations that overlapped with the distribution of the participant's occipital pain. The leads were then connected to an external pulse generator, and a waterproof bandage was placed over the lead exit sites. Stimulation consisted of biphasic, asymmetric, charge balanced pulses delivered at 100 Hz with amplitudes between 0.2 and 30 mA and pulse widths between 10 and 200 μsec. Participants controlled stimulation using a wireless Bluetooth remote, and leads were left indwelling for up to 60 days before removal by a study investigator using gentle traction.

### 2.4 Outcomes & assessments

The study's primary endpoint evaluated the proportion of participants with clinically significant reductions (≥30%) in average pain intensity and/or pain interference (using BPISF-5 and -9, respectively) at the end of treatment, as previously reported [20]. The key effectiveness outcome in the present analysis was the proportion of participants reporting substantial (≥50%) relief from average pain intensity and/or pain interference. Average pain intensity was quantified as the mean score across a seven-day pain diary.

Secondary outcomes included average pain intensity in other regions of the head and neck, neck pain-related disability (evaluated using the Neck Disability Index, NDI), the impact of headache on daily life (evaluated using the Headache Impact Test, HIT-6), and Patient Global Impression of Change (PGIC). The NDI (a modification of the Oswestry Disability Index [ODI]) scoring ranges from 0 to 50 and measures the severity of disability due to neck pain [21]. Clinically significant improvement in neck disability was defined as a 5-point reduction in NDI from baseline [21]. The HIT-6 is a validated metric for the impact of headache on daily life and other activities and is scored between 36 and 72 [22]. Clinically significant improvement in headache disability was defined as a 5-point reduction in HIT-6 from baseline [22]. PGIC measures the global change from baseline experienced by a participant using a 7-point scale. Scores range from −3 (“Very much worse”) to 3 (“Very much improved”) with 0 indicating “No change”; a score ≥1 (at least “Minimally improved”) was considered clinically significant.

Assessments were performed at key time points including baseline (i.e., prior to the start of treatment [SOT]), lead withdrawal at 60 days (end of treatment [EOT]), and at prospectively defined follow-ups: 3 months, 6 months, 9 months, and 12 months from SOT. Device safety was assessed by study-related adverse events (AEs).

### 2.5 Statistical analysis

Because the goals of this study were to evaluate the feasibility and safety of percutaneous 60-day PNS targeting the occipital nerves, data are presented descriptively. A power analysis was not performed to define the cohort size, and *a priori* statistical comparisons were not defined. Continuous variables are reported as mean ± standard error (se) unless otherwise specified. Categorical variables are reported as proportions with 95% confidence intervals (CI). Weekly recall pain scores (BPISF-5) were collected at each follow-up visit and were used to impute missing diary data if needed. An independent, third-party biostatistics group (WCG, Princeton, NJ) performed analyses on primary (pain intensity, pain interference) and secondary (HIT-6, PGIC, NDI, Headache Days) outcomes and prespecified multiple imputation using predictive mean matching to account for missing data. Multiple imputation was performed for missing data at 3, 6, 9, and 12 months from the start of treatment (there were no missing data prior to the 3-month visit). A worst-case sensitivity analysis (i.e., missing values imputed as treatment non-responders) was also performed.

Primary and secondary endpoints are presented for the full analysis set with imputation unless otherwise stated, and as-observed data are available in the supplementary materials. A per protocol population was also prospectively defined as participants who received two leads bilaterally and completed the EOT pain diary, but both participants who failed to meet these criteria withdrew from follow-up. The per protocol and full analysis datasets are therefore identical at 12 months in the as-observed outcomes and there are only marginal differences in the imputed datasets; therefore, the per protocol dataset is not presented separately in this report.

All data were stored on a secure clinical data management platform (Zelta, Merative, Ann Arbor, MI). Each site's principal investigator approved the data for their respective site. All co-authors, including the study sponsor, had access to the final data and approved the final manuscript.

## 3 Results

### 3.1 Study population

**Fig. 1:**
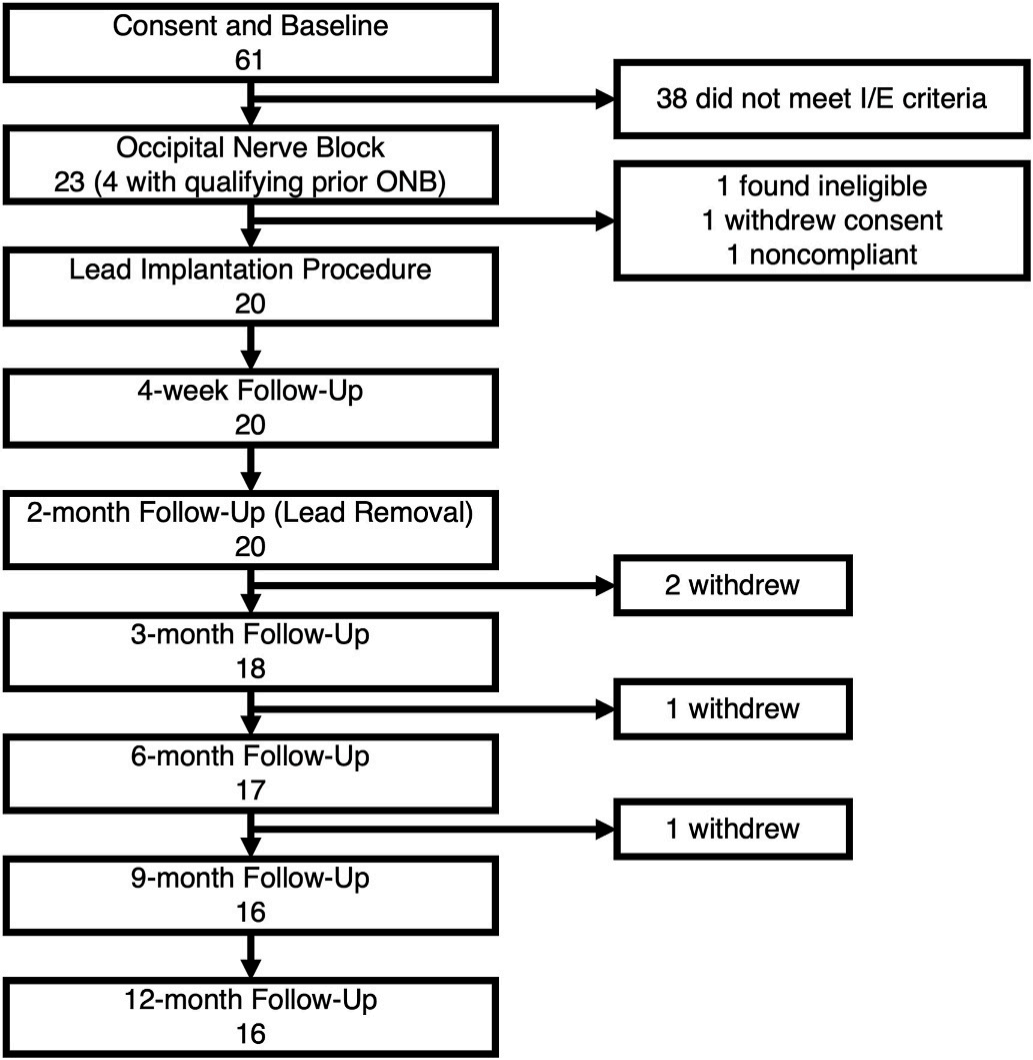
Consolidated Standards of Reporting Trials (CONSORT) diagram. Occipital nerve block (ONB) was performed at least 7 days prior to lead implantation procedure unless the participant had an eligible block prior to enrollment. The treatment period spanned from Lead Implantation Procedure to 2-month Follow-up (Lead Removal). Abbreviation: I/E, inclusion/exclusion.

Twenty participants were enrolled in this study cohort between October 2022 and March 2024 as previously reported (Fig. 1) [20]. The study cohort represented a mix of diagnoses and included participants with CGH (50%, n = 10/20), ON (25%, n = 5/20), and participants with characteristics of both (25%, n = 5/20). Of the 20 participants who enrolled, all were followed through 2 months (i.e., EOT), and 16 were followed through 12 months (4 withdrew). Across the 7 participating sites, no one site enrolled more than 25% of the total study population (enrollment size by site ranged from 1 to 5 participants).

At baseline, participants rated their average occipital pain intensity and pain interference as 5.8 ± 0.2 and 6.4 ± 0.3 out of 10, respectively. Seventy-five percent (n = 15/20) reported severe impact of headache on daily life at baseline (HIT-6 ≥ 60), and 4 additional participants reported substantial impact (HIT-6 ≥ 56). Ninety percent (n = 18/20) reported moderate to severe pain-related neck disability (NDI ≥15). Participants had commonly failed multiple other therapies prior to the study including non-opioid pain medications (95%, n = 19/20), physical therapy (85%, n = 17/20), chiropractic treatment or spinal manipulation (65%, n = 13/20), and others (Table 1). All participants had previously used at least 2 other treatment types for their headache prior to enrollment, and a majority (60%, n = 12/20) had undergone at least 7 prior treatment types, indicating highly treatment-refractory headache among the majority of study participants.

**Table 1:** Demographic and baseline information for enrolled participants (N = 20). Data are represented as mean (se, range) for continuous variables and count (%) for categorical variables. Abbreviations: se, standard error; TENS, transcutaneous electrical nerve stimulation. ^†^Includes non-opioid analgesics or other medications impacting pain (e.g., anticonvulsants, antidepressants, muscle relaxants). Some categories are slightly higher than the initial study publication due to the subsequent identification of additional previous therapies in two participants.

Participant Demographics	Value
Age (years; mean, range)	54.9 (3.7, 29-81)
Body Mass Index (mean, range)	28.7 (1.3, 19-38)
Sex (F:M)	14:6
Duration of Headache Pain (years; mean, range)	5.4 (1.5, 0.3-27.2)
**Headache Diagnosis**	
Cervicogenic Headache	10 (50%)
Occipital Neuralgia	5 (25%)
Both Cervicogenic Headache and Occipital Neuralgia	5 (25%)
**Work Status at Baseline**	
Employed (Full Time)	12 (60%)
Part Time Work	1 (5%)
Retired	5 (25%)
Disabled	1 (5%)
Leave of Absence	1 (5%)
**Previous Conservative Therapies**	
Non-opioid pain medications	19 (95%)
Physical therapy	18 (90%)
Chiropractic treatment/spinal manipulation	13 (65%)
Injections (nerve block and/or corticosteroid)	13 (65%)
Massage therapy	13 (65%)
Acupuncture/dry needling	12 (60%)
Opioid pain medications	12 (60%)
Exercise therapy	11 (55%)
TENS	10 (50%)
Radiofrequency ablation	8 (40%)
Botox injections	4 (20%)
**Medications In Use at Baseline**	
Opioid pain medications	6 (30%)
Non-opioid pain medications^†^	17 (85%)
Over-the-counter pain medications (e.g., aspirin, ibuprofen, acetaminophen, etc.)	16 (80%)

### 3.2 Durability of relief from pain and pain interference

**Fig. 2:**
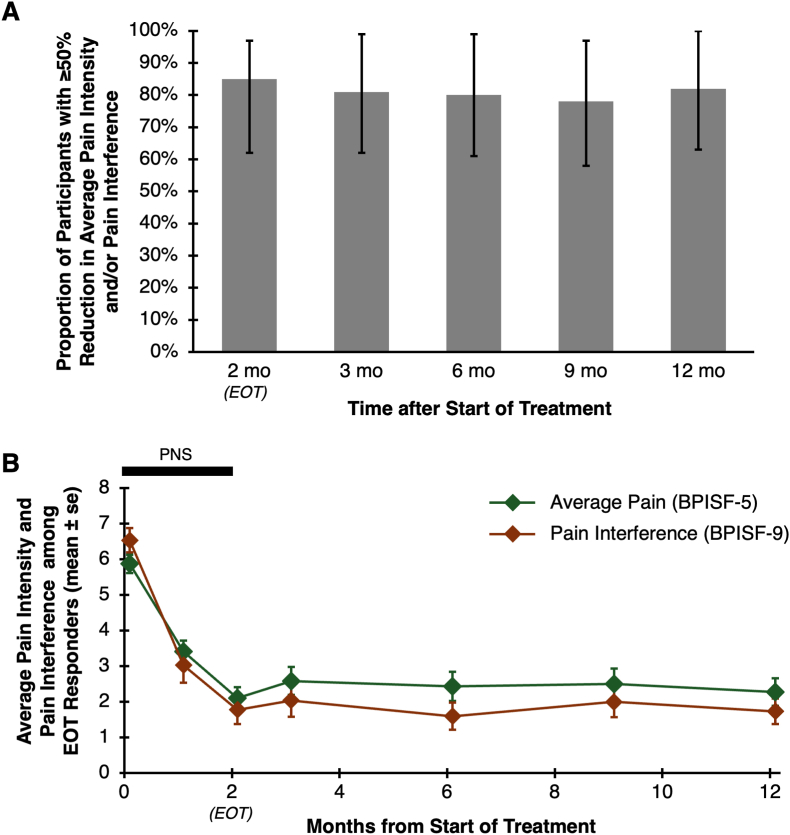
Reductions in average pain and pain interference. A) Proportion of participants with ≥50% reduction in average pain intensity and/or pain interference (±95% confidence intervals, n = 20) B) Mean average pain and pain interference among EOT responders from baseline through 12 months (n = 17). Abbreviations: BPISF, Brief Pain Inventory Short Form; EOT, end of treatment; mo, months; PNS, peripheral nerve stimulation; se, standard error.

At 12 months, 82% of participants (95% CI: [63-100%], n = 20 for full analysis set with imputation) reported sustained and substantial reduction (≥50%) in pain intensity and/or pain interference, which was comparable to the treatment response rate at EOT (85%, 95% CI: [62-97%], Fig. 2A). Of the 4 participants who withdrew from the study, 2 were responders at the last follow-up visit (one at EOT and one at 3-month follow-up) while the remaining 2 were non-responders (one at EOT and one at 6-month follow-up). A sensitivity analyses of the response rate at 12 months that accounted for a worst-case scenario (i.e., participants with missing data considered non-responders at 12-mo) was within the 95% CI bounds (65% and [63-100%], respectively). The responder rate in the full analysis dataset with imputation differed by ≤ 5% from the as observed data for all timepoints, including at 12 months where the as-observed response rate was 81% (95% CI: [54-96%], n = 13/16, Supplementary Fig. 1). All participants completing the 12-month follow-up (n = 16/16, 100%) also reported ≥30% reductions in average pain and/or pain interference. Individual participant's values for pain intensity and pain interference are plotted in Supplementary Fig. 2 alongside as-observed mean values for the full analysis set.

The mean reductions in pain and pain interference in all participants at 12 months were 59% ± 8% and 70% ± 7%, respectively (Fig. 2B). Among responders to the PNS treatment (≥50% reductions in pain and/or pain interference at 2 months (EOT)), the sustained reductions in average pain intensity and pain interference at 12 months were 61% ± 7% and 73% ± 5%, respectively (Fig. 2B). The mean percent reductions in average pain and pain interference scores in the full analysis dataset with and without imputed values were within 5% at each time point supporting the notion that the as-observed data were representative of the full study population.

### 3.3 Secondary outcomes

**Fig. 3:**
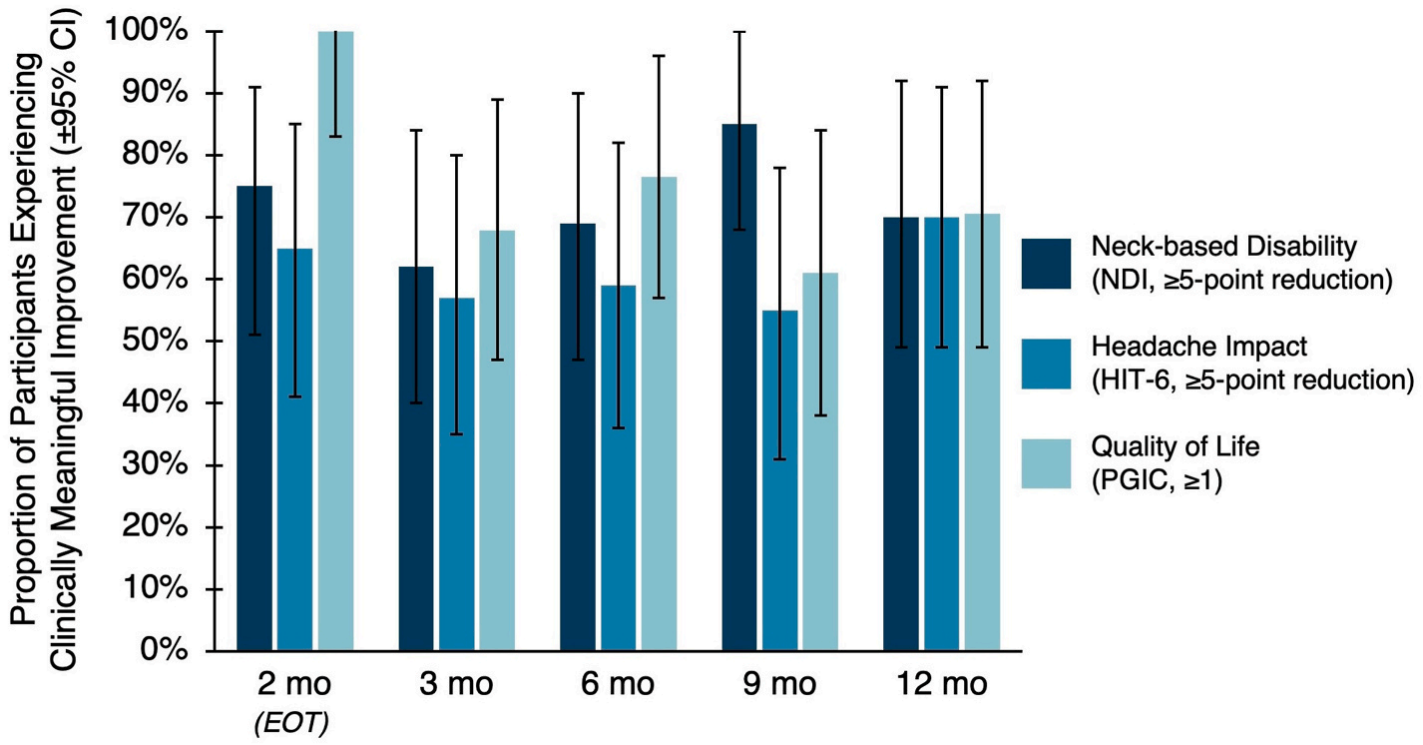
Proportion of participants experiencing clinically meaningful improvements in secondary outcomes: pain-related neck disability (measured using Neck Disability Index [NDI], ≥5-point reduction compared to baseline), headache impact on daily life (measured using Headache Impact Test [HIT-6], ≥5-point reduction compared to baseline), and quality of life (measured using Patient Global Impression of Change [PGIC] ≥1). EOT = end of treatment; CI = confidence interval. Abbreviations: mo, months.

Participants showed sustained improvements in pain-related neck disability (NDI), headache impact (HIT-6), quality of life (PGIC), and headache days per week at 12-month follow-up compared to baseline values (Table 2). The mean NDI score decreased from 22.3 ± 1.5 at baseline to 12.7 ± 1.2 at 12 months. On average, participants had severe impact of headaches on their daily life at baseline based on HIT-6 severity definitions (62.7 ± 1.0) which decreased to “some” impact at 12 months (51.3 ± 2.3) [23]. Based on definitions of clinically meaningful improvement, neck disability (70%, 95% CI: [49-92%]) and headache impact (70%, 95% CI: [49-91%]) were meaningfully reduced in a majority of participants at 12 months (Fig. 3).

**Table 2:** Secondary outcomes. Data presented as mean ± se. EOT = end of treatment.

Time Point	Neck Disability Index (NDI)	Headache Impact Test (HIT-6)	Patient Global Impression of Change (PGIC)	Headache Days Per Week
Baseline	22.3 ± 1.5	62.7 ± 1.0	N/A	5.5 ± 0.4
2 months (EOT)	13.0 ± 1.4	53.7 ± 1.9	1.6 ± 0.1	3.5 ± 0.6
3 months	13.7 ± 1.5	54.9 ± 1.7	1.2 ± 0.2	3.6 ± 0.7
6 months	14.4 ± 1.7	53.2 ± 2.2	1.2 ± 0.2	4.0 ± 0.7
9 months	13.1 ± 1.6	54.7 ± 2.4	1.0 ± 0.3	2.9 ± 0.6
12 months	12.7 ± 1.6	51.3 ± 2.3	1.2 ± 0.3	2.5 ± 0.6

The percent change in mean headache days (i.e., a day with at least one headache recorded in the pain diary) decreased from 5.5 ± 0.4 at baseline to 2.5 ± 0.6 through 12 months across all participants (a 55% reduction), indicating participants experienced an additional 3 headache-free days per week on average.

At EOT, all participants (100%) reported improvements in quality of life (at least minimally improved, PGIC≥1) with a majority reporting sustained improvement through 12 months (71%, 95% CI: [49-92%], Fig. 3).

### 3.4 Additional head and neck regions

**Fig. 4:**
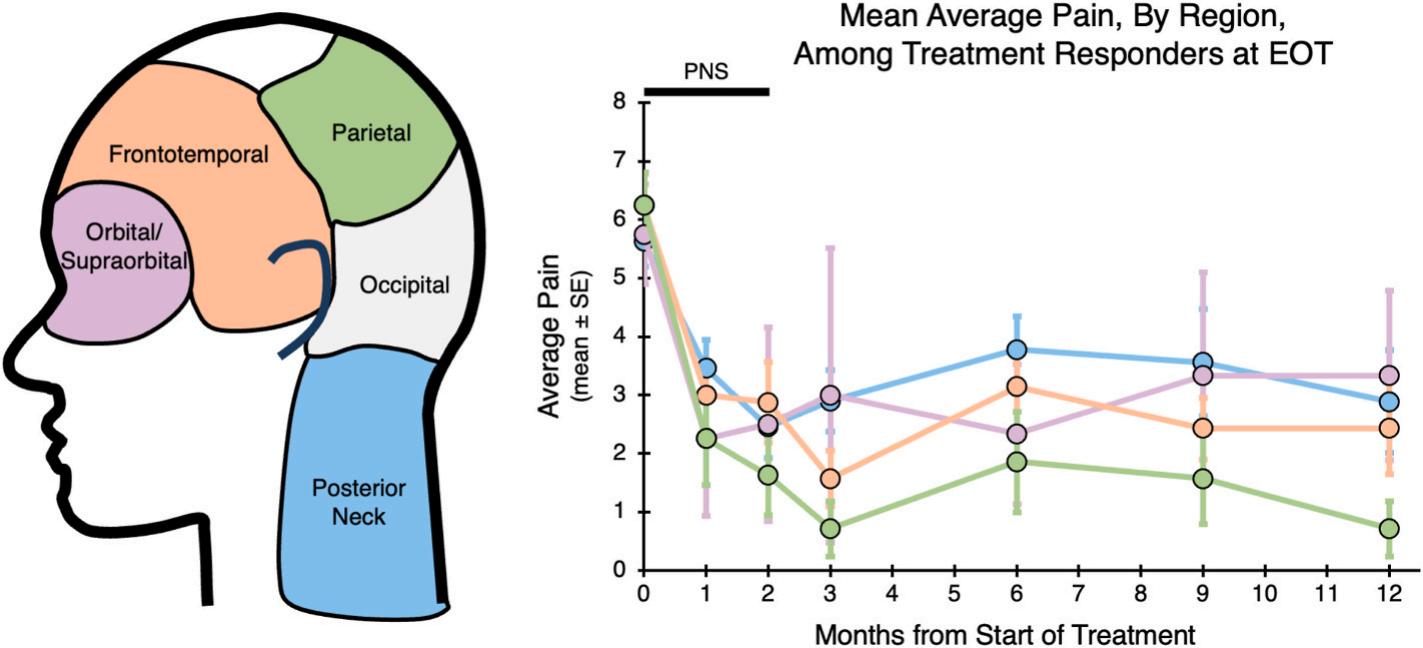
Average pain among responders in other areas of the head and neck. Mean pain intensity is shown among participants who met the EOT responder definition (≥50% reduction in average pain and/or pain interference) and also had at least 4/10 pain at baseline in a given region. Head regions are color coded for visual representation and consist of Posterior Neck (blue, n = 11 at baseline, n = 9 at 12 months), Orbital/Supraorbital (purple, n = 4 at baseline, n = 3 at 12 months), Frontotemporal (orange, n = 8 at baseline, n = 7 at 12 months), and Parietal (green, n = 8 at baseline, n = 7 at 12 months). (For interpretation of the references to color in this figure legend, the reader is referred to the Web version of this article.)

While leads were implanted in the posterior cervical region to target occipital nerves, the study also evaluated pain in other areas of the head and neck in addition to participants’ occipital pain. As an exploratory outcome, these outcomes are reported as observed and were not included in the imputation model. A majority of participants reported average pain ≥4/10 in at least one additional region at baseline (85%, n = 17/20). Across these areas, participants who responded to treatment of their occipital pain (see Fig. 2) also reported that their average pain in other head regions was consistently reduced at EOT with durable reductions in pain through 12 months (Fig. 4). For example, the mean average parietal pain (i.e., pain immediately cephalad to the occipital region) score among participants with at least moderate pain in that region at baseline decreased from 6.3 ± 0.6 at baseline (n = 8) to 0.7 ± 0.5 at 12 months (n = 7), representing an 88% reduction.

### 3.5 Other treatments

Through 12 months, a majority of participants did not pursue additional interventions (e.g., steroid injection, radiofrequency ablation) after EOT (69%, n = 11/16). Eleven participants followed through 12 months had used an intervention for their headache prior to enrollment, and a majority (64%, n = 7/11) discontinued the use of interventions through the follow-up period. One participant who had not previously used an intervention did not respond to PNS treatment and utilized interventions during follow-up. Of the five participants who received additional intervention during the follow-up period, a majority (n = 3/5) were non-responders at 12 months, indicating the additional treatments likely did not impact their study outcomes. Two other participants were prescribed additional medications during the follow-up period, both of which were non-opioid anticonvulsant medications.

As reported previously, six participants were using opioid medications at baseline of whom four reduced their consumption at EOT with a moderate mean reduction (16%) in milligram morphine equivalent (MME) [20]. Four of those participants were followed through 12 months. Three of the four participants whose pain medication diaries indicated opioid use at least in part for headache at baseline continued taking opioids at 12 months but no longer noted headache as the reason. One participant continued taking opioids for their headache at 12 months but decreased their daily MME consumption by 33% compared to baseline (7.7 MME/day and 5.1 MME/day, respectively). No patients followed through 12 months initiated new opioid medication usage.

Medication use for headaches was quantified using the 7-day medication diary. Compared to baseline, a majority of participants had fewer days where they took medications to control their headache at 12 months compared to baseline (56%, n = 9/16), and six of those participants had complete cessation of headache medications (100% reduction). Six participants reported no change in medication frequency, and one participant increased medication use from 3 days/week to 7 days/week. In summary, almost all participants (94%, n = 15/16) reported either a reduction or no change in daily medication use at 12 months compared to baseline.

### 3.6 Safety

No serious or unanticipated study-related adverse events (AE) occurred among this study cohort. All study-related AEs were non-serious and followed to resolution. The most common AEs reported were mild skin irritation or itching due to bandage adhesion. The AEs related to the percutaneous 60-day PNS in this study were reported in a prior publication in which data were complete through 3 months [20], and no new device-related adverse events were reported during the follow-up presented here through 12 months.

## 4 Discussion

The present study with follow-up outcomes through 12 months from the start of percutaneous 60-day PNS supports the potential use of this treatment to provide significant and sustained reductions in pain intensity and pain interference for individuals with refractory cervicogenic headache or occipital neuralgia. Overall, 82% of participants reported substantial (≥50%) relief from pain and/or pain interference through 12 months which highlights the durability of the responses initially observed at EOT (85%). These long-term outcomes are supported by a strong safety profile observed in this participant cohort including no serious or unanticipated device effects, no lead fractures, and no additional AEs reported in the follow-up period after lead removal [20]. As a non-pharmacological, minimally-invasive, and non-destructive treatment option, the ability of 60-day PNS to produce robust, sustained improvements without permanent implantation creates an opportunity for neuromodulation to be employed earlier in the treatment algorithm for occipital headache.

In addition to disabling pain, patients with chronic occipital headache commonly experience dramatic impairment in other dimensions of health including quality of life, function, and sleep [24,25]. Effective and sustained treatment of pain can facilitate long-lasting improvements in functional domains, leading to changes that break this cycle and enable an exit from the chronic pain state [26]. In the present study, through 12 months, 71% of participants reported improvement in QoL (PGIC ≥1) and 70% reported clinically meaningful reductions in headache impact (HIT-6 reduction ≥5). These results indicate that 60-day PNS for pain may support other multidimensional improvements, allowing patients to live, work, sleep, and socialize with reduced burden of headache.

The present study also highlights an overlap of occipital and cervical pain among patients with CGH and ON. Posterior neck was the most common concurrent pain area; 65% (n = 13/20) of participants had average posterior neck pain ≥4/10 at baseline, and 90% (n = 18/20) reported at least moderately severe neck disability (NDI ≥15) at baseline. Prior studies have identified strong relationships between neck pain and headache in patients with CGH and ON whereby periods of intense neck pain are temporally tied to headaches or neck pain can act as a trigger for headaches [27,28]. Pain outside the occipital region (including the neck) may also be referred due to the interconnectedness of neural circuits, including between the occipital nerves and the trigeminocervical complex [1,2,29]. Participants with baseline posterior neck pain ≥4 reported an average decrease from 5.6 ± 0.4 to 2.5 ± 0.5 at EOT. Additionally, 75% [95% CI: 51-91%] of all participants reported clinically important change in NDI (≥5 point decrease) at EOT, providing evidence that 60-day PNS may provide relief not only in the distribution of the target occipital nerves, but also in other distinct areas that may be susceptible to referred pain. These improvements in neck disability were sustained, with a majority of participants reporting a meaningful decrease of NDI scores compared to baseline through 12 months.

In addition to the neck, CGH and ON-related pain can also occur in or be referred to other areas of the head, including the orbital/supraorbital, frontotemporal, and parietal regions. Participants with at least moderate (≥4/10) pain in each region reported mean decreases in pain intensity at EOT and during follow-up (Fig. 3). These reductions may be a result of reduced referred pain originating in the occipital region, or modulation of trigeminocervical networks.

This study demonstrates the potential for sustained headache relief through 12 months using PNS leads implanted for up to 60 days. Previous studies investigating stimulation of the occipital nerves for various headache disorders have shown promising signs of efficacy but utilized permanently implanted systems not designed for implantation in the head and neck [5,30]. In one randomized controlled trial (RCT) investigating permanently implanted spinal cord stimulation leads used for occipital nerve stimulation in patients with chronic migraine, 47.8% of participants in the active study group reported at least 50% reduction and/or monthly headache days at 52 weeks (i.e., about 12 months) [8]. Other studies investigating the use of permanently implanted systems have similarly reported significant pain relief in patients with various headache conditions ranging from migraine to cluster headache and occipital neuralgia [5,31,32]. In the current cohort, a high proportion of participants reported substantial improvements in pain and reductions in headache impact and disability without the need for permanently implanted systems, which patient preference research has suggested may be desirable to many patients [33,34]. This study complements existing clinical and healthcare economic evidence suggesting the use of percutaneous 60-day PNS treatment may provide sustained benefit through one year [33,35].

Other prospective studies investigating the use of percutaneous 60-day PNS for chronic pain conditions, such as shoulder pain [36], knee pain [13], neuropathic pain following amputation [15], and low back pain [14], have reported similarly durable treatment effects. Additionally, recent follow-up survey studies after 60-day PNS for low back and shoulder pain found that pain relief can be sustained through up to 4-5 years which facilitated improvements in functional and quality of life domains [37,38]. The unique ability of this short-term PNS treatment to produce long-term pain relief has been proposed to derive from the robust selective activation of targeted fibers within the stimulated nerve (e.g., the occipital nerves in the present study) and subsequent reconditioning of centralized chronic pain features [39], and recent evidence suggests these effects may be consistent across varying degrees of pain chronicity [13,40,41].

## 5 Limitations

The present prospective single-arm study enrolled 20 participants to determine the feasibility of implanting 60-day PNS leads targeting the occipital nerves and evaluate the long-term effectiveness and safety of system. A control group was not included in the study design, as the goal of the study was to establish feasibility, with regard to effectiveness and safety, of 60-day PNS used for CGN and ON. Although a direct comparison with other treatments and/or interventions was not made, participants had on average tried multiple prior treatments including physical therapy, injections, and/or opioid medications (Table 1), indicating their head pain was refractory to multiple attempts at symptom control prior to study enrollment. While the study lacked a control group, the treatment effects were in line with prior studies investigating 60-day PNS for various pain conditions, including multiple placebo-controlled and standard of care-controlled RCTs [12,14,15]. These promising results warrant a subsequent controlled trial investigating the use of 60-day PNS for CGH and ON in a larger study population to increase confidence in the treatment effects observed in the present study.

Although the inclusion of two diagnoses (i.e., CGH and ON, determined by their presence in the participants’ records only) may have introduced heterogeneity to the study population, all participants had pain primarily in the areas innervated by the occipital nerves that could not be attributed to other headache disorders (e.g., tension-type headache, analgesic medication over-use or withdrawal headache, cluster headache, vascular and non-vascular intracranial disorders, migraine), and these results suggest that 60-day PNS was effective across the included head pain types. While 4 of the 20 enrolled participants withdrew from follow-up prior to 12 months, a robust statistical analyses using multiple imputation and other sensitivity analyses added confidence to the estimates of sustained treatment effects. Prior to applying statistical imputation, weekly recall pain scores were collected at each visit and used to impute missing diary data. As previously reported, less than 2% of diary data points through 3 months were imputed in this manner [20], and no pain diaries were missing or incomplete from still-enrolled participants at 6-12 months so no additional pain diary imputation was required.

Additionally, participants were permitted to receive other treatments for their pain after the end of the 60-day PNS treatment to balance study objectives and participant retention with ethical considerations of restricting treatment during follow-up. Other interventions in the follow-up period could interact with long-term outcomes after PNS, however a minority of participants reported pursuing other interventions (n = 5). Because 3 of the 5 participants with additional interventions were already characterized as non-responders at 12 months, the impact of these events on responder rates and study conclusions is potentially minimal.

## 6 Conclusion

The development of PNS devices better suited for mobile areas of the body like the head and neck has opened new opportunities for effective, non-pharmacological management of headache. In the present feasibility study, participants with CGH or ON had sustained relief from pain and pain interference with resulting improvements in function and reductions in disability at 12-month follow-up following a percutaneous 60-day PNS treatment. Participants had no new device-related adverse events reported following the 3-month follow-up. These findings support the clinical feasibility for 60-day PNS in the treatment of refractory pain due to CGH or ON. Future studies may incorporate controls and explore this procedural approach in other primary and secondary headache disorders.

## Funding

This study was supported by SPR.
