# Supplementary material for: Sustained relief of chronic headache using percutaneous 60-day peripheral nerve stimulation treatment: 12-month results from a multicenter, prospective clinical study
Source: Interv Pain Med. 2026 Jul 30;5(3):100806. doi: 10.1016/j.inpm.2026.100806 (PMC13449970; doi:10.1016/j.inpm.2026.100806)
Supplement: Supplementary file 1 [file mmc1.docx]

# Supplementary Materials

*Supplementary Figure 1. Reductions in average pain and pain interference among the as observed full analysis set and dataset including imputation. A) Proportion of participants with ≥50% reduction in average pain intensity and/or pain interference (±95% confidence intervals) B) Mean average pain and pain interference among responders at baseline to 12 months. Imputation data shown for months 3-12 as month 3 was the earliest timepoint with imputed data. Abbreviations: BPISF, Brief Pain Inventory Short Form; EOT, end of treatment; mo, months; PNS, peripheral nerve stimulation; se, standard error.*

*Supplementary Figure 2. As-observed data for Average Pain Intensity (BPISF-5, A) and Pain Interference (BPISF-9, B) for all individual participants (gray) and the overall average (black, mean±se).*
